# Case of necrotic enteritis associated with campylobacteriosis and coccidiosis in an adult Indian peacock (*Pavo cristatus*)

**DOI:** 10.1186/s12917-022-03260-1

**Published:** 2022-05-02

**Authors:** Aleksandra Ledwoń, Małgorzata Murawska, Izabella Dolka, Dorota Chrobak Chmiel, Piotr Szleszczuk

**Affiliations:** 1 Department of Pathology and Veterinary Diagnostics of the Institute of Veterinary Medicine, Warsaw University of Life Sciences, Nowoursynowska 159c, 02-776 Warsaw, Poland; 2 Department of Preclinical Sciences, of the Institute of Veterinary Medicine, Warsaw University of Life Sciences, Nowoursynowska 159c, 02-776 Warsaw, Poland

**Keywords:** Peafowl, *Campylobacter jejuni*, *Eimeria pavonina*, Necrotic enteritis

## Abstract

**Background:**

To date, *Campylobacter jejuni* has not been found to be pathogenic to peafowl. The available publications show that out of a total of 44 samples tested from peafowl, this bacterium was isolated only in two cases. *Eimeria pavonina* infestations in the peafowl have been described, but no fatal cases have been reported yet.

**Case presentation:**

The four-year-old peacock was presented with chronic diarrhea, emaciation and weakness. Post mortem examination revealed enlarged and pale kidneys, small intestinal mucosal necrosis and thickening of intestinal wall, and pericardial effusion. The histopathological examination revealed necrotic enteritis with marked mononuclear cells infiltration associated with the presence of coccidia, additionally there was histological evidence of septicemia in liver and kidneys. Bacteria identification was based on light microscopy of the small intestine sample, culture, and biochemical tests. Further identification was based on PCR. Antimicrobial susceptibility profile was created by determination of minimal inhibitory concentration (MIC) values for 6 antimicrobial agents from 5 different classes. PCR assays were performed to detect virulence factors genes responsible for motility, cytolethal distending toxin production, adhesion and internalization. Bacteriology of the small intestine sample showed abundant growth almost exclusively of *Campylobacter jejuni*, resistant to ciprofloxacin, gentamycin and ampicillin. Bacteria was sensitive to Amoxicillin + clavulanic acid, tetracycline, and erythromycin. All tested virulence factors genes have been detected. The parasitological examination was performed by microscopic examination of fresh faeces and intestinal content, and revealed the moderate number of *Eimeria pavonina*, *Histomonas meleagridis*, single *Capillaria* spp. eggs as well *Heterakis* spp. like parasites.

**Conclusion:**

The above case shows that a virulent isolate of *Campylobacter jejuni* in combination with a parasitic invasion may cause chronic enteritis in peafowl, which most likely led to extreme exhaustion of the host organism and death.

**Supplementary Information:**

The online version contains supplementary material available at 10.1186/s12917-022-03260-1.

## Background

### Campylobacteriosis

*Campylobacter jejuni* (*C. jejuni*) is relatively often isolated from chickens [1, 2] and is considered as non-pathogenic for these birds, however there are reports of hepatitis in poultry (known as avian vibrionic hepatitis - AVH), caused by this bacterium [3] in the presence of risk factors (e.g. stress, immunosuppressive conditions of the host) [2]. The source of *Campylobacter* spp. infection for birds is carrier faeces [4].

*C. jejuni* is of interest to veterinarians mainly due to its zoonotic potential [5].

In humans, this infection is a common cause of bacterial enteritis, but can be associated also with Guillian-Barré syndrome (GBS), reactive arthritis and necrotic *enterocolitis* in children [6, 7].

In birds the pathogenicity of *C. jejuni* depends on its origin and the age. According to some authors, isolates from humans are more pathogenic, for newly hatched chickens than chicken-origin isolates [8]. Screening studies on healthy 31 Indian peafowl from three Michigan zoos have not shown the presence of *Campylobacter* spp., while a moderate number of coccidia has been found in these birds [9]. In a study conducted by a laboratory in Louisiana, *C. jejuni* was found in one Indian peafowl out of 10 samples tested from these birds [10]. Research by Misawa et al. [11] in zoo animals showed the presence of *C. jejuni* in one of the 3 studied peacocks.

### Coccidiosis

Coccidia invasions in peafowl have been reported by several authors in the past. Among others, the following species of coccidia in peafowl have been described in Asian countries and Egypt: *Eimeria pavonina* [12], *Eimeria mandalin* [13], *Eimeria roscoviensis* [14], *Eimeria mayurai* [15], *Eimeria riyadhae*, *Eimeria arabica* [16] and *Eimeria pavoaegyptica* [17]. In Pakistan, coccidial oocysts were found in 20–30% of peafowl faecal samples [18]. In peafowl kept in Europe, *Isospora mayuri* and *Eimeria pavonina (E. pavonina)* were reported [19, 20].

### *Campylobacter* spp. and *Eimeria* spp. coinfection

Invasion of *Eimeria tenella*, which is closely related to *E. pavonina* [20], has been confirmed to increase *C. jejuni* colonization in the intestines of chickens [21]. To date, no fatal co-infection of *C. jejuni* and *E. pavonina* in the Indian peafowl has been reported.

## Case presentation

In June 2020, a 4-year-old Indian peacock (*Pavo cristatus*) has been brought to the Veterinary Clinic of the Institute of Veterinary Medicine, Warsaw University of Life Sciences due to weakness and chronic diarrhea. The peacock was a private property of a person who had 4 more peahens, that did not show any signs of disease. Peafowl were free range. The bird had atrophy of the pectoral muscles and was unable to move independently. The owner’s reported that weakness and diarrhea were observed in this bird 3 months ago and numerous coccidia and *Histomonas meleagridis* were detected at the microscopic examination of the fresh faeces sample. Transient improvement was obtained after the use of toltrazuril (Baycox 2.5%, Bayer, Germany) at a dose 7 mg / kg of body weight, followed by ronidazole (Trichonidazole, Biovet Puławy, Poland) at a dose of 60 mg /1 l of drinking water for 7 days. The condition of the peacock, however, gradually began to deteriorate, and bird died.

**Fig. 1:**
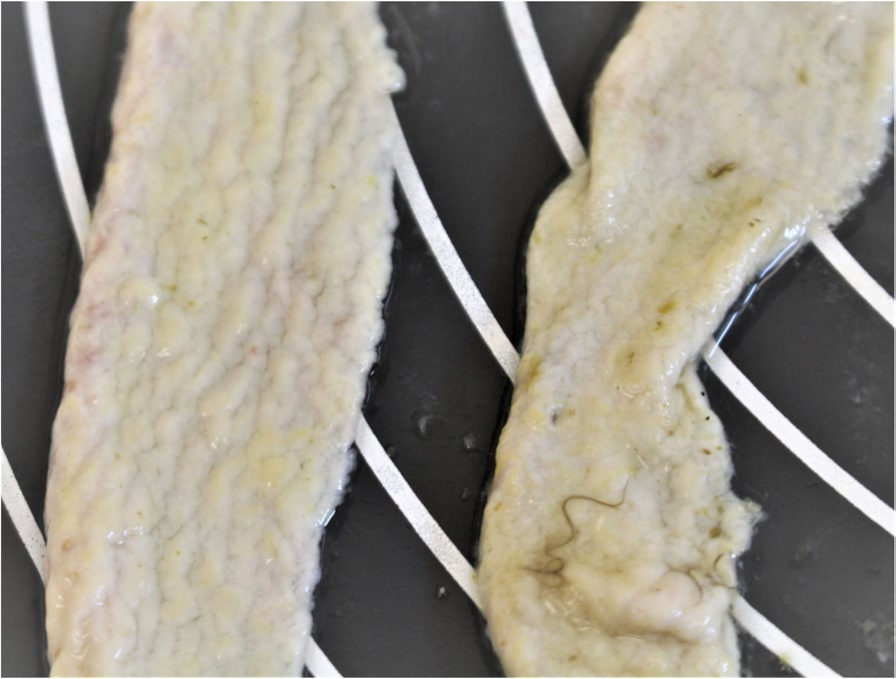
Mucosal thickening of the jejunum with prominent folds, covered with a pale yellow coating

Necropsy was performed on the same day. Necropsy showed that the peacock was emaciated (Supplementary Fig. 1), the feathers around the cloaca were soiled with diarrheal faeces (Supplementary Fig. 2). Serous fluid in the pericardial sac was found. The testes were inactive and kidneys were moderately enlarged and pale. The mucosa of the small intestine was significantly thickened and covered with pale yellow coating (Fig. 1). The lumen of the caeca was dilated, but mucosa of the caeca was unchanged (Supplementary Figs. 3–5). Only the proximal caecum and rectum were thickened and pale pink in color (Supplementary Fig. 5). Macroscopically, no changes were found in other organs.

### Histopathology

**Fig. 2:**
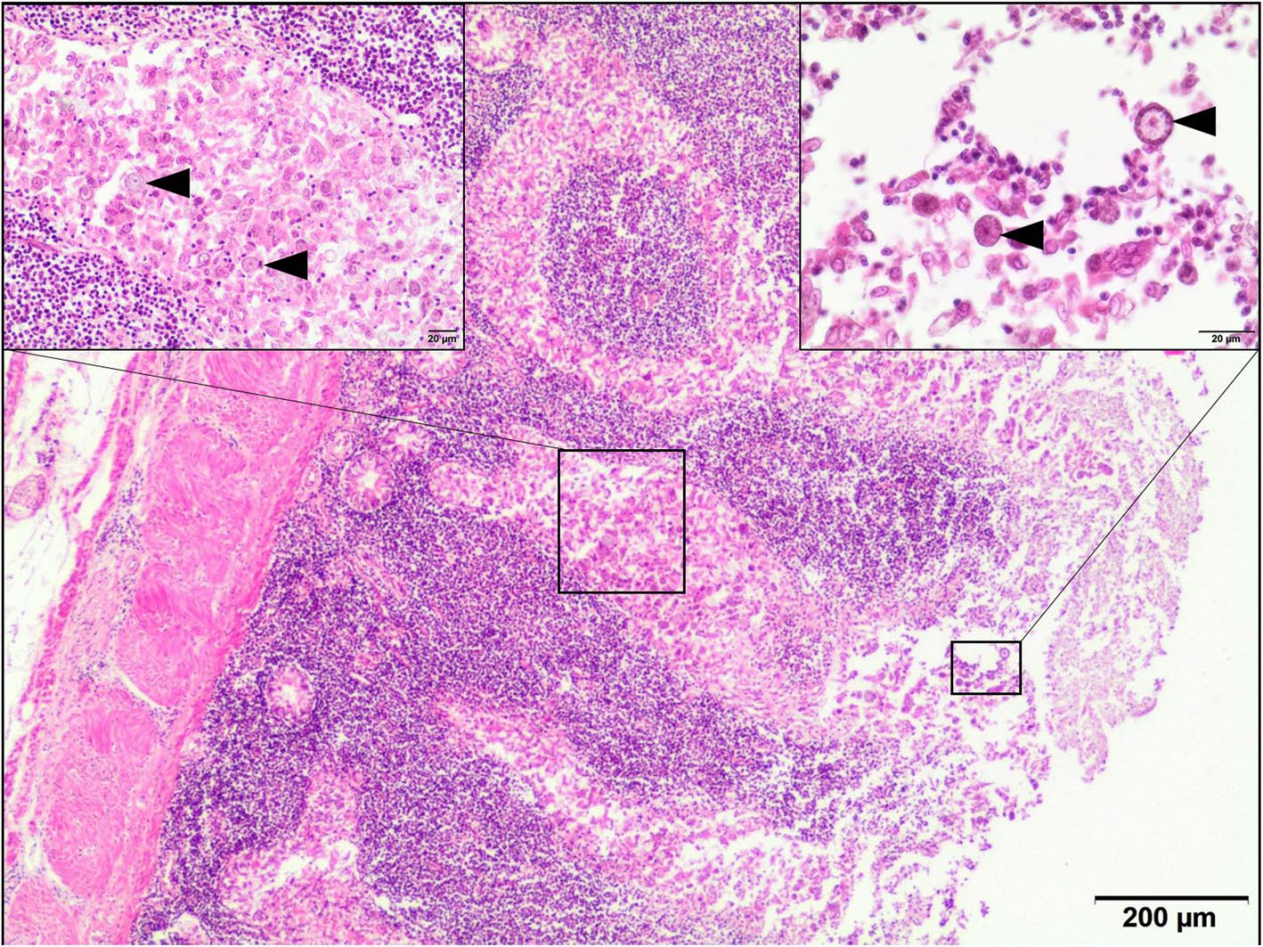
Intestine. Necrotic enteritis. Marked inflammation mainly consisted of mononuclear cells in the lamina propria of the mucosa; perivascular inflammation in the muscular membrane. Villus atrophy, crypt epithelial cell proliferation and necrosis, congestion. H-E, 40x. Left insert: Destroyed enterocytes of the intestinal crypt due to invasion of coccidia (arrowheads), 200x. Right insert: free coccidian parasites (arrowheads) intermixed with exfoliated epithelial cells on the destroyed luminal surface of the villi. 400x.*

**Fig. 3:**
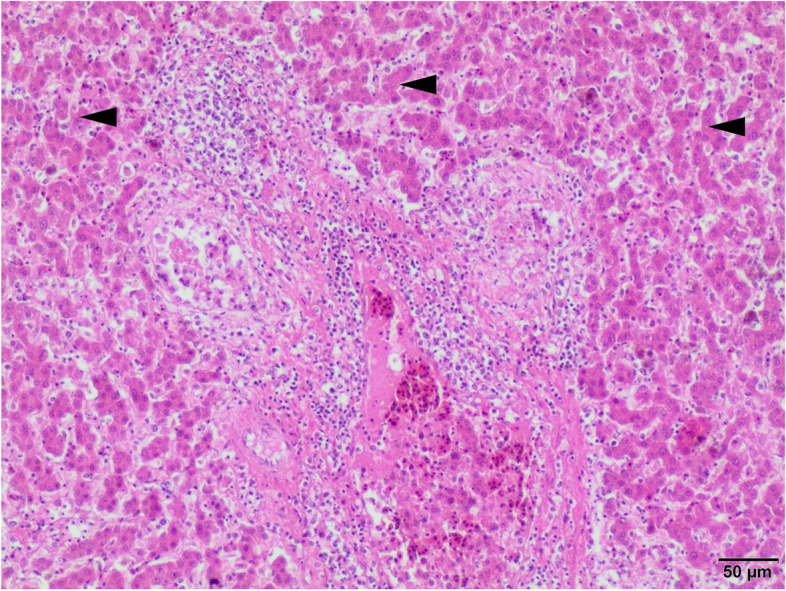
Liver. Massive inflammation of the portal triad. Liver parenchyma displays necrotic and degenerated hepatocytes (microvacuolar fatty change; arrowheads), venous thrombi, and congestion. H-E, 100x

**Fig. 4:**
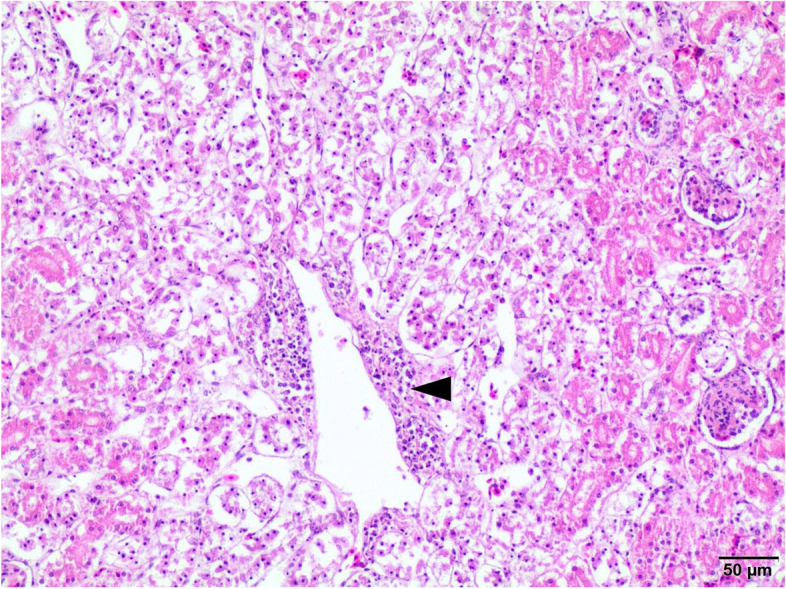
Kidney. Massive perivascular inflammation (arrowhead), proliferative glomerulopathy, necrosis of renal epithelial cells both in the cortex and medulla. H-E, 100x

Tissue samples (liver, kidney, and intestines) were fixed in 10% neutral-buffered formalin, dehydrated in increasing gradients of ethyl alcohol and embedded in paraffin. The tissue sample was then cut in the microtome at four micron thickness. Finally, paraffin sections were stained with haematoxylin and eosin (H-E). In the jejunum: massive, diffuse inflammatory infiltrate mainly composed of mononuclear cells (numerous lymphocytes, plasma cells, macrophages), intermixed with coccidian parasites in the increased lamina propria showing marked architectural distortion. Severe destruction of the mucosa: loss of the villi (blunt or flattened), marked epithelial necrosis, and sloughing, the loss or damaged crypts, moderate congestion of the mucosa, and focal small grains structures in blood vessels resemble bacterial clusters were detected (Fig. 2). In addition, perivascular mononuclear cell infiltration in the muscular and serous membranes was present focally. In the liver multifocal necrosis of hepatocytes and microvacuolar fatty degeneration of hepatocytes, disintegrated areas with fibrinoid necrosis of vessels surrounded by inflammatory cells (mainly mononuclear cells), fibrin thrombi, numerous were found (Fig. 3). In the kidneys: perivascular mononuclear inflammatory infiltrate, necrosis of blood vessel walls, and necrosis of tubular epithelial cells, glomerulonephritis, fibrin thrombi in the capillary of the glomerulus were found (Fig. 4).

### Parasitology

In order to detect the presence of parasites from the content of the small intestine, caecum and rectum, direct wet mount and stained preparations were made using the Ziehl-Neelsen staining and Hemacolor® rapid staining (Merck, Germany). To determine coccidia species identity, DNA was isolated from the content of the jejunum using the QIAamp DNA Stool Mini Kit (Qiagen GmbH, Germany), following a two-day protocol [22], additionally introducing sample homogenization with glass beads for 10 min on the GeneReady homogenizer (Hangzhou Lifereal Biotechnology Co., Ltd., China). We amplified 767 base pairs of the cox-1 mitochondrial gene using universal *Eimeria* spp. primer pairs described by Miska et al. [23]. The PCR product was then sequenced. Examination of the rectal and small intestine contents showed an average 4 coccidia oocyst in the high-dry power field (400x magnification), and two eggs of *Capillaria* spp. in the preparation. Examination of the caecal content revealed large, round, mobile flagellates with the morphology of *Histomonas meleagridis* and several nematodes similar to *Heterakis gallinarum*. In the preparation stained with the Ziehl-Neelsen stain, average of 3 coccidia oocysts were found per high-power field (1000x magnification). The presence of *Histomonas meleagridis* was confirmed in a microscopic slide, stained with the Hemacolor® method. Based on the sequencing of the PCR product, the coccidia were identified as *Eimeria pavonina*, and its sequence was uploaded to GenBank and assigned accession number: OM891494.

#### Bacteriology

##### Isolation and identification

A fragment of the jejunum taken aseptically was used for direct microscopic examination and for culturing. A direct microscope slide was stained using Gram-staining method. After the analysis of direct microscopic slide collected clinical material was streaked on Columbia Agar plates with 5% sheep blood (CBA; GRASO Biotech, Poland) and on modified charcoal-cefoperazone-deoxycholate agar plates (mCCDA; GRASO Biotech, Poland), followed by the streak plate method was performed. Another 2 fragments of the small intestine were placed in the sterile tubes containing 5 mL of Preston Broth with Preston Modified Supplement (BIOCORP, France) and 5% defibrinated sheep blood (GRASO Biotech, Poland). Agar plates were incubated at 42 °C under microaerophilic conditions created by GasPak Campy Container System (BD, USA) for 48 h. Preston’s broth/s were incubated as described above but with shaking (120 RPM) and after pre-propagation 100 μL of the liquid media was streaked on the CBA and mCCDA plates and incubated as described above, without shaking. Obtained colonies were streaked eventually on the CBA plates and incubated as described above. Preliminary identification was based on colony morphology, both on Columbia Agar and mCCDA plates, Gram staining, motility, microscopic morphology, catalase and oxidase tests. Further identification, to the species level, was conducted by PCR [24]. Briefly, genomic DNA was extracted using Genomic Mini isolation kit (A&A Biotechnology, Poland), following the manufacturer’s protocol with minor modifications.

For the identification of *Campylobacter jejuni* (mapAF, mapAR for mapA target – 604 bp amplicon)) and *Campylobacter coli* (Mu3, Mu for Random target – 364 bp amplicon) species using the PCR method, two pairs of species-specific primers [24] and also, genomic DNA’s of standard strains (*C. jejuni* 81–176 and *C. coli* 605) and sterile, deionized water were used as positive and negative controls respectively.

##### Antimicrobial susceptibility testing

Antimicrobial susceptibility profile was created by determination of minimal inhibitory concentration (MIC) values, using ETEST® gradient strips (Biomerieux, France). Six antimicrobial agents from five classes were tested (Tables 1 and 2). Choice of antimicrobial agents was based on their usage in veterinary medicine and the necessity of monitoring resistance of *C. jejuni* to antimicrobials used in human treatment due to zoonotic nature of campylobacteriosis. Interpretation of the obtained results was based on EUCAST [25] or CLSI [26] guidelines Table 1 summarizes information on used antimicrobial agents and criteria of interpretation. Additionally, for ciprofloxacin resistance mechanism determination, a 270 bp fragment of the *gyrA* gene was amplified, according to Chatur et al. [27] and sent for Sanger sequencing to Genomed (Poland) and analyzed for point mutations in our laboratory using DNA Baser Assembler software v. 5.11.3 (Heracle BioSoft SRL, Romania).

**Table 1:** List of antimicrobial agents, their abbreviations and concentrations used for creating antimicrobial susceptibility profile. Interpretation according to: ^a^EUCAST, ^b^CLSI

Antimicrobial agent class	Antimicrobial agent	Abbr.	ETEST	Interpretation		
			Concn. [mg/L]	S	I	R
**β-lactams**	Ampicillin^a^	AM	0,016–256	≤2	x	≥8
	Amoxicillin + clavulanic acid^a^	AMC	0,016–256	≤2	x	≥8
**Aminoglycosides**	Gentamicin^a^	GE	0,016–256	≤0,5	x	≥0,5
**Tetracyclines**	Tetracycline^b^	TE	0,016–256	≤4	8	≥16
**Macrolides**	Erythromycin^b^	E	0,016–256	≤8	16	≥32
**Fluoroquinolones**	Ciprofloxacin^a^	CIP	0,002–32	≤0,5	x	≥0,5

**Table 2:** Antimicrobial agents susceptibility profile of *Campylobacter jejuni* isolate

Antimicrobial agent class	Antimicrobial agent	Abbr.	MIC value	Interpretation
**β-lactams**	Ampicillin	AM	256	R^a^
	Amoxycyllin + clavulanic acid	AMC	0,5	S^b^
**Aminoglycosides**	Gentamicin	GE	0,75	R
**Tetracyclines**	Tetracycline	TE	0,75	S
**Macrolides**	Erythromycin	E	1	S
**Fluoroquinolones**	Ciprofloxacin	CIP	3	R

^a^R-resistant

^b^S- sensitive

##### Virulence factors genes detection

PCRs were performed to detect chosen virulence factors genes responsible for: motility (*flaA*, *flaB*), cytolethal distending toxin production (*cdtA*, *cdtB*, *cdtC*), adhesion and invasion to the host’s cells (*ciaB*, *pldA*, *cadF*, *flpA*). Assays were performed on genomic DNA of the identified *C. jejuni* isolate (isolation as described in “Isolation and identification” section), using primers and conditions as in reference publications. Used primers along with the amplicons sizes are listed in Table 3.

**Table 3:** Primers used for *C. jejuni* virulence factors genes detection by PCR

Target gene	Primer name	Amplicon size [bp]	Sequence 5′ – 3′	Ref.
*flaA*	flaA-F	1728	GGATTTCGTATTAACACAAATGGTGC	[28]
	flaA-R		CTGTAGTAATCTTAAAACATTTTG	
*flaB*	fB1	260	AAGGATTTAAAATGGGTTTTAGAATAAACACC	[29]
	fA2		GCTCATCCATAGCTTTATCTGC	
*cdtA*	cdtA-f	370	CCTTGTGATGCAAGCAATC	[30]
	cdtA-R		ACACTCCATTTGCTTTCTG	
*cdtB*	cdtB-F	620	CAGAAAGCAAATGGAGTGTT	[31]
	cdtB-R		AGCTAAAAGCGGTGGAGTAT	
*cdtC*	cdtC-F	182	TTGGCATTATAGAAAATACAGTT	[31]
	cdtC-R		CGATGAGTTAAAACAAAAAGATA	
*ciaB*	ciaB-F	527	TGCGAGATTTTTCGAGAATG	[32]
	ciaB-R		TGCCCGCCTTAGAACTTACA	
*pldA*	pldA-F	385	AAGAGTGAGGCGAAATTCCA	[32]
	pldA-R		GCAAGATGGCAGGATTATCA	
*cadF*	cadF-F2B	400	TTGAAGGTAATTTAGATATG	[33]
	cadF-R1B		CTAATACCTAAAGTTGAAAC	
*flpA*	Cj1279c-F	832	TCAGAAGATGGCAAGGTTATAGAAG	[34]
	Cj1279c-R		GTTATTGCTATTGATTCAGCTGGAC	

#### Bacteriology results

##### Isolation and identification

A direct microscope slide from small intestine sample stained with Gram method showed numerous Gram-negative, thin, helical rods with almost no other biota (Supplementary Fig. 6). After incubation on mCCDA plates (direct inoculation), medium-numerous, medium-sized, round, flat, grey colonies with no gloss were obtained in pure culture. After pre-propagation on the supplemented Preston Broth, on Columbia Blood Agar plates we obtained growth of the pure culture of medium-sized, round, flat-convex, greyish and non-haemolytic colonies. Grown bacterial colonies were both catalase and oxidase positive. Microscopic slide from microbial cultures on blood agar plates, stained with Gram method, showed Gram-negative, thin, helical rods. Wet-mount slide from blood agar culture showed thin, motile, helical rods with characteristic corkscrew-like movement.

As a result of the PCR assay with mapAF and mapAR primers, we obtained single amplification product, about 600 bp in size during the electrophoresis in 1% agarose gel in 1X TAE buffer, the same as for positive control, which allowed us to identify isolate as *Campylobacter jejuni*, which sequence was uploaded to GenBank and assigned accession number OM927984. We obtained no product with Mu3 and Mu4 primers (for *C. coli* identification).

#### Antimicrobial agents susceptibility of *Campylobacter jejuni* isolate

Additionally, as a result of sequencing of the fragment of *gyrA* gene, point mutation (transition) in position 257 (257C > T) was found, resulting in amino acid substitution in codon 86 (Thr-86-Ile), which is the most common and frequent fluoroquinolones resistance mechanism among *Campylobacter* genus [35].

##### Virulence factors genes detection

We have detected all of the selected genes responsible for motility (*flaA*, *flaB*), cytolethal distending toxin production (*cdtA, cdtB, cdtC*) and adhesion and internalization process (*ciaB, pldA, cadF, flpA*) by obtaining single product of expected size (Table 3) in each PCR assay.

## Discussion and conclusions

Necrotic enteritis in poultry is a disease caused mainly by *Clostridium perfringens* [36]. Other bacterial, parasitic, and viral factors have also been reported to cause similar changes in poultry, but this was not *Campylobacter* spp. [37]. Previous studies [9–11] show that, *C. jejuni* is not often isolated from peafowl. So far, no case of a peafowl disease caused by this bacteria has been described. However, in other bird species, *C. jejuni* was isolated from the intestinal tract of clinically affected and asymptomatic birds [2, 4]. Clinical signs of avian campylobacteriosis have been observed in pet birds (mainly *Passeriformes*) and generally were associated with subacute to chronic hepatitis, include lethargy, anorexia, diarrhea and emaciation [4]. At necropsy, the liver is enlarged, pale or greenish, congested, with or without hemorrhage. Coalescing necrotic hepatitis is a common histological finding [4].

In poultry, *Campylobacter jejuni* has been considered as a commensal microorganism which colonizes its primary host rather than infecting it, in the absence of any obvious clinical signs, however, recent studies show possible pathogenicity of this bacterium for chickens [38]. The clinical signs of campylobacteriosis were experimentally induced in young chickens. Infected birds showed symptoms of diarrhea, weight loss, and even died [39].. Microscopic changes were found, ranging from moderate infiltration of mononuclear cells in ileum and cecum [39], to villus atrophy in the jejunum [40], mucosal damage, notably thickening, shortening and fusion of villi in the ileum [41]. In the presented case, the changes in the small intestine were similar, and their greater advancement may be caused by the concomitant parasitic invasion.

In the examined peacock, no changes in the liver were found macroscopically, but microscopic examination showed advanced necrosis and inflammation in the liver and kidneys. It is known that toxins produced by *C. jejuni* may be responsible for necrotic changes in the chicken embryo liver [42].

Antibiotics used in *Campylobacter* infections include macrolides such as erythromycin [43], tetracyclines, streptomycin and furans [8]. The isolate from the studied case additionally showed sensitivity to amoxicillin with clavulanic acid. The resistance of the tested strain to ciprofloxacin determined by single point mutation in the gyrA gene, is in line with the recent trends in fluoroquinolone resistance in strains of members of the genus *Campylobacter,* isolated from livestock and clinical samples from several countries [33].

So far, the authors have not observed fatal cases of coccidiosis in adult peafowl and no mortality cause by coccidia in peafowl has been documented in available literature. The only description of *E. pavonina* infestation reported in Europe [20] is a case of marked depression in a young peacock (during winter), while other 33 adult and young peafowl in this place, showed no symptoms of the disease [20]. Interestingly, no parasites were detected in faecal samples from the diseased bird, but in samples of birds from the same and other aviaries [20]. In presented case, although many oocysts were not found in the faeces and intestinal contents, the infestation was confirmed by histopathological examination.

Toltrazuril is a triazinetrione derivative administered orally in the drinking water for the treatment of coccidiosis in chickens and turkeys. The recommended dose and duration of treatment for chickens and turkeys is 7 mg/kg bw per day for two consecutive days (https://www.ema.europa.eu/en/medicines/veterinary/referrals/toltrazuril)- and this is how the described peacock was treated 3 months before his death. In the case described by Hauck et al. [20], treatment with toltrazuril was at the same dose, but twice for 3 days with a break of 5 days. Studies conducted by Gesek et al. [44] with doses of 7 mg / kg bw, 14 mg /kg bw. and 24.5 mg / kg for 2 days in Japanese quails, showed that only a dose of 24.5 mg / kg bw, led to total destruction of the coccidia, but only in two of the six treated birds. However, the use of such high doses causes pathologic toxic changes in the liver and kidneys [44]. Other available drugs that may be used in the treatment of coccidiosis in ornamental *Gallinaceous* birds are sulfonamides. Studies in turkeys have shown that toltrazuril is more effective than sulfonamides [45]. There is therefore a need to test the effectiveness of other triazine compounds, such as aminomizuril and ethanamizuril [46] in peafowl.

In the presented case, *Histomonas meleagridis* was found in the cecum, but no changes typical of this invasion were observed. Much more often cryptosporidiosis was diagnosed as the cause of changes in the intestines in peacocks [47, 48], but in the case described, these parasites were not found in microscopic examination.

Presented case shows that despite the fact that *Campylobacter jejuni* is considered non-pathogenic for most healthy chickens [3], it may induce clinical signs and mortality in peafowl. This case provide guidance to veterinarians who struggle with chronic diarrhea in peafowl, to include campylobacteriosis in diagnostic tests, as well as do not neglect coccidiosis therapy even in adult birds.

## Supplementary Information

**Additional file 1.**

**Additional file 2.**

**Additional file 3.**

**Additional file 4.**

**Additional file 5.**

**Additional file 6.**

## Data Availability

The data generated or analyzed during this study are included in this published article and its supplementary files. The raw data of DNA- sequencing are available from the NCBI database under accession number PRJNA819941. (SRX14610090 and SRX14609836) https://www.ncbi.nlm.nih.gov/sra/?term=PRJNA819941.

## Abbreviations

mg / kg.: Milligrams per kilogram
mg /l.: Milligrams per liter
spp.: Species
μl: Microliter
bp: Base pair
PCR: Polymerase chain reaction
bw: Body weight
ppm: Parts per million
