# Supplementary figures and images for: Case of necrotic enteritis associated with campylobacteriosis and coccidiosis in an adult Indian peacock (Pavo cristatus)
Source: BMC Vet Res. 2022 May 2;18:160. doi: 10.1186/s12917-022-03260-1 (PMC9063363; doi:10.1186/s12917-022-03260-1)

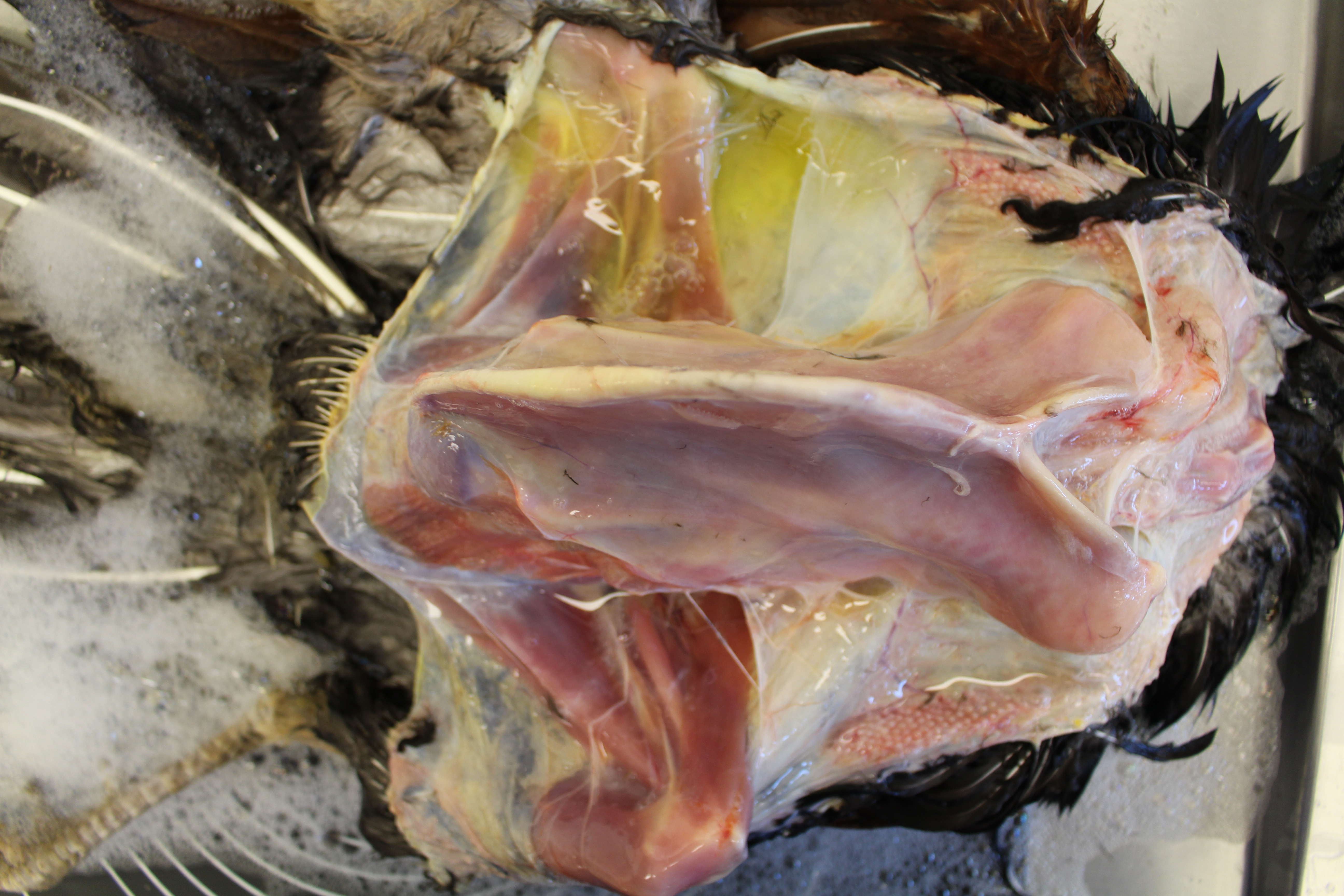

Supplement: Supplementary file 1 — Additional file 1. [file 12917_2022_3260_MOESM1_ESM.jpg]

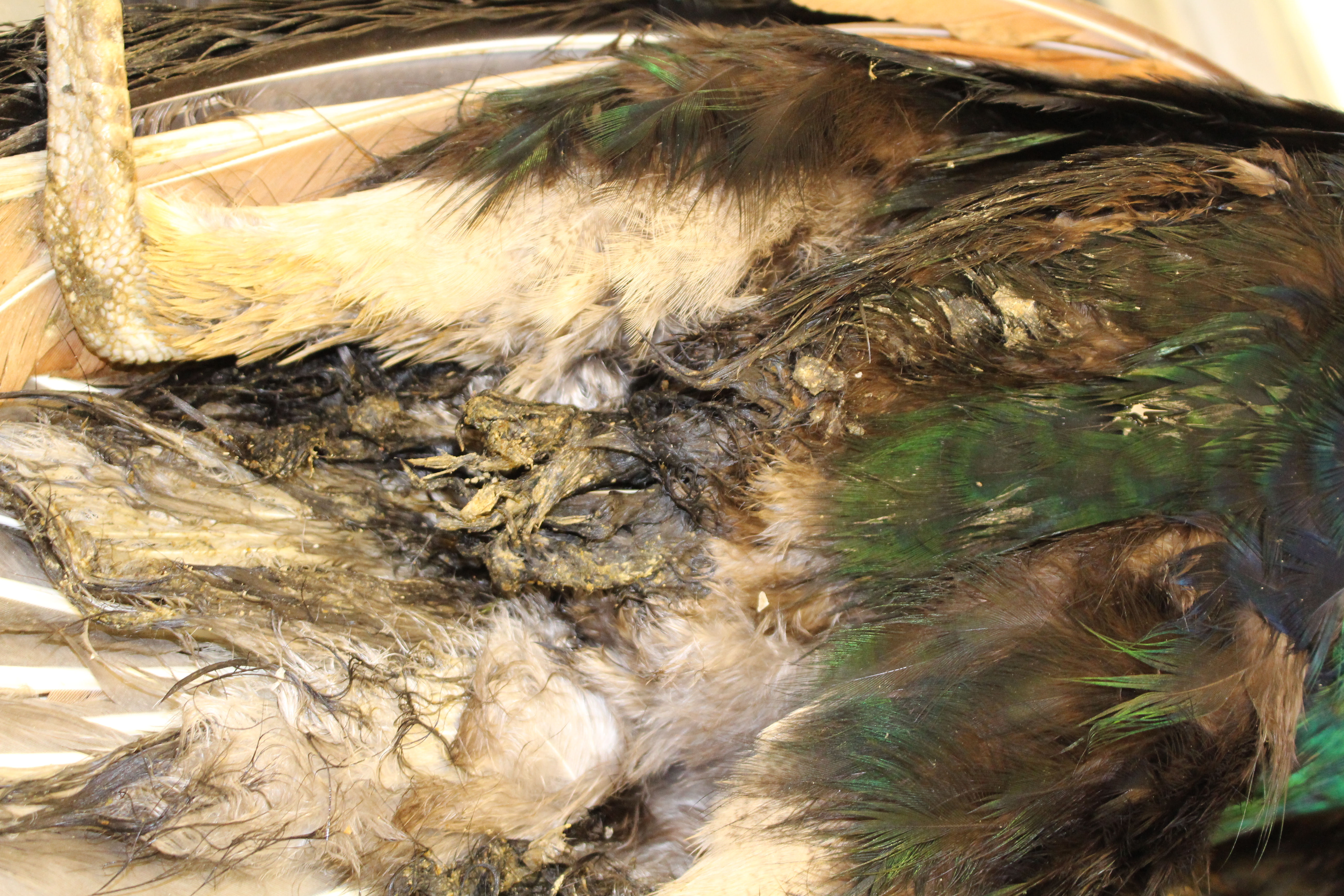

Supplement: Supplementary file 2 — Additional file 2. [file 12917_2022_3260_MOESM2_ESM.jpg]

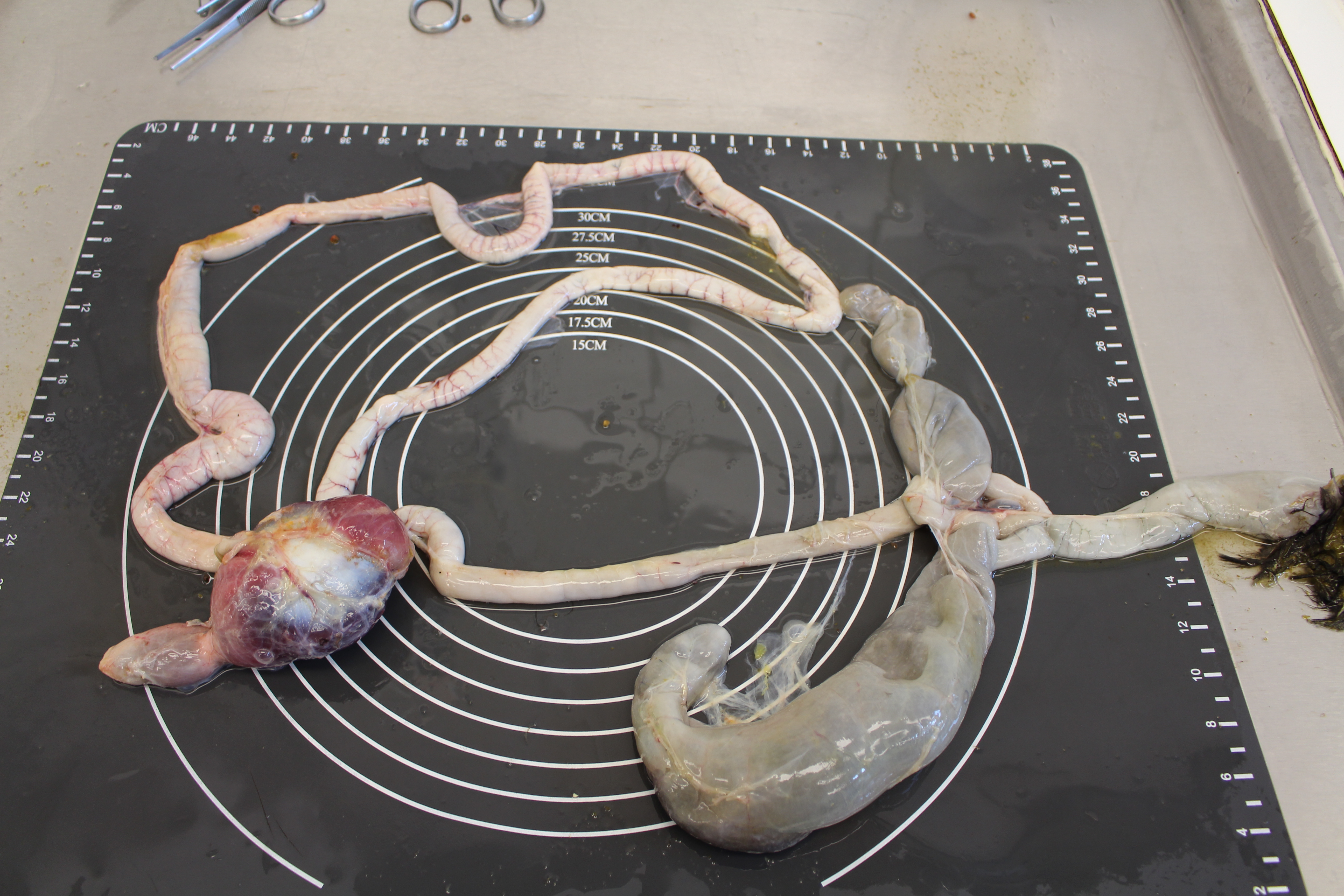

Supplement: Supplementary file 3 — Additional file 3. [file 12917_2022_3260_MOESM3_ESM.jpg]

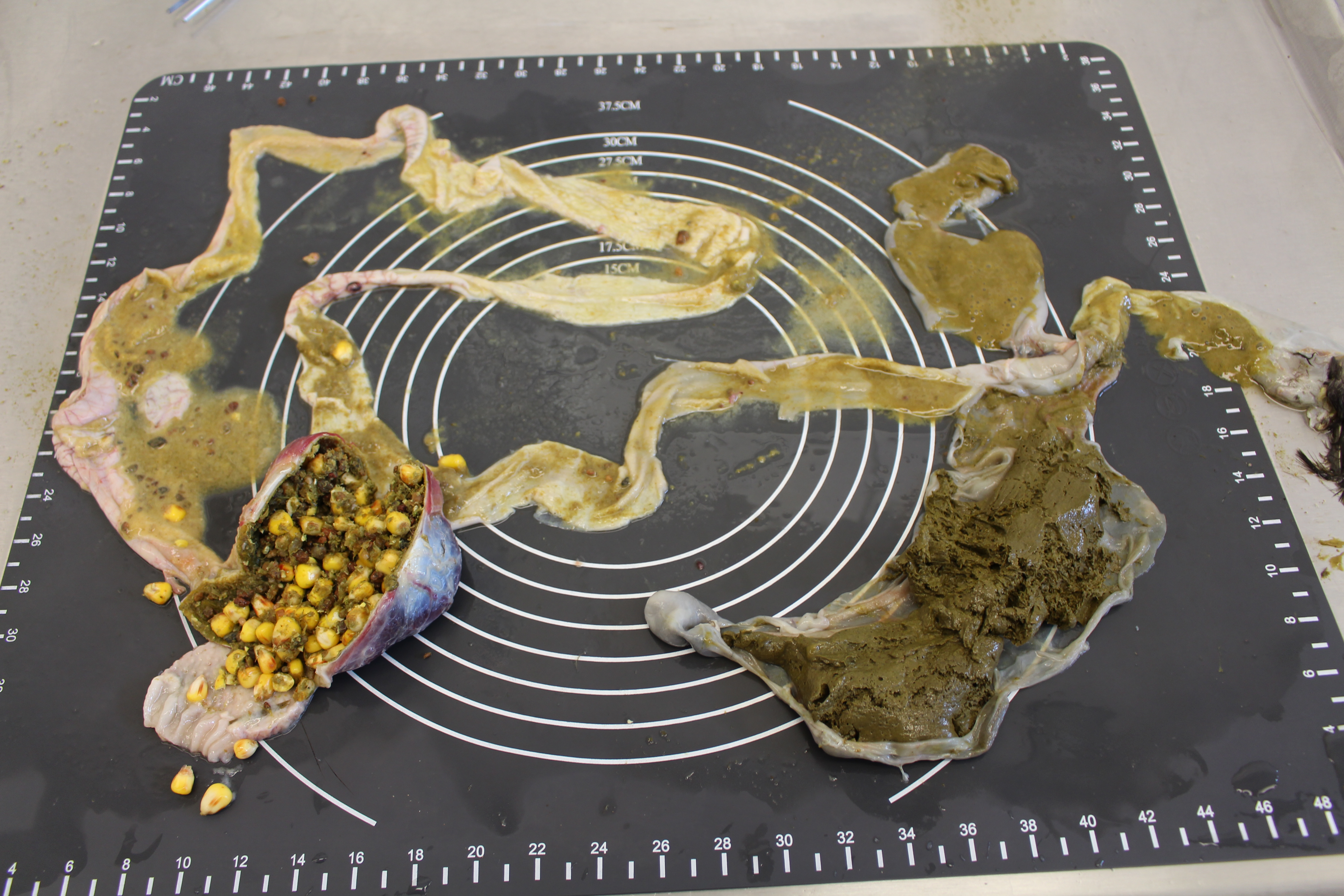

Supplement: Supplementary file 4 — Additional file 4. [file 12917_2022_3260_MOESM4_ESM.jpg]

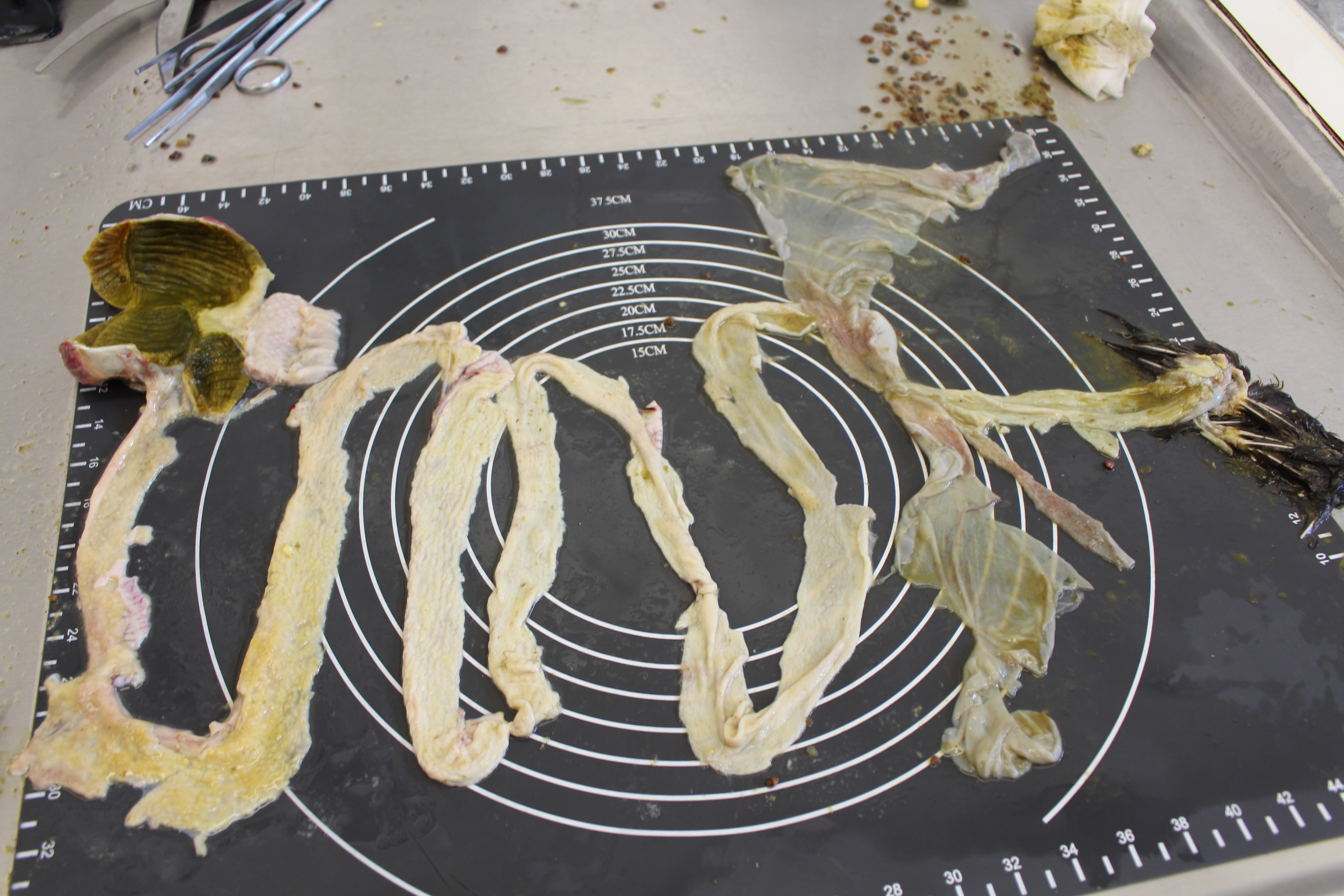

Supplement: Supplementary file 5 — Additional file 5. [file 12917_2022_3260_MOESM5_ESM.jpg]

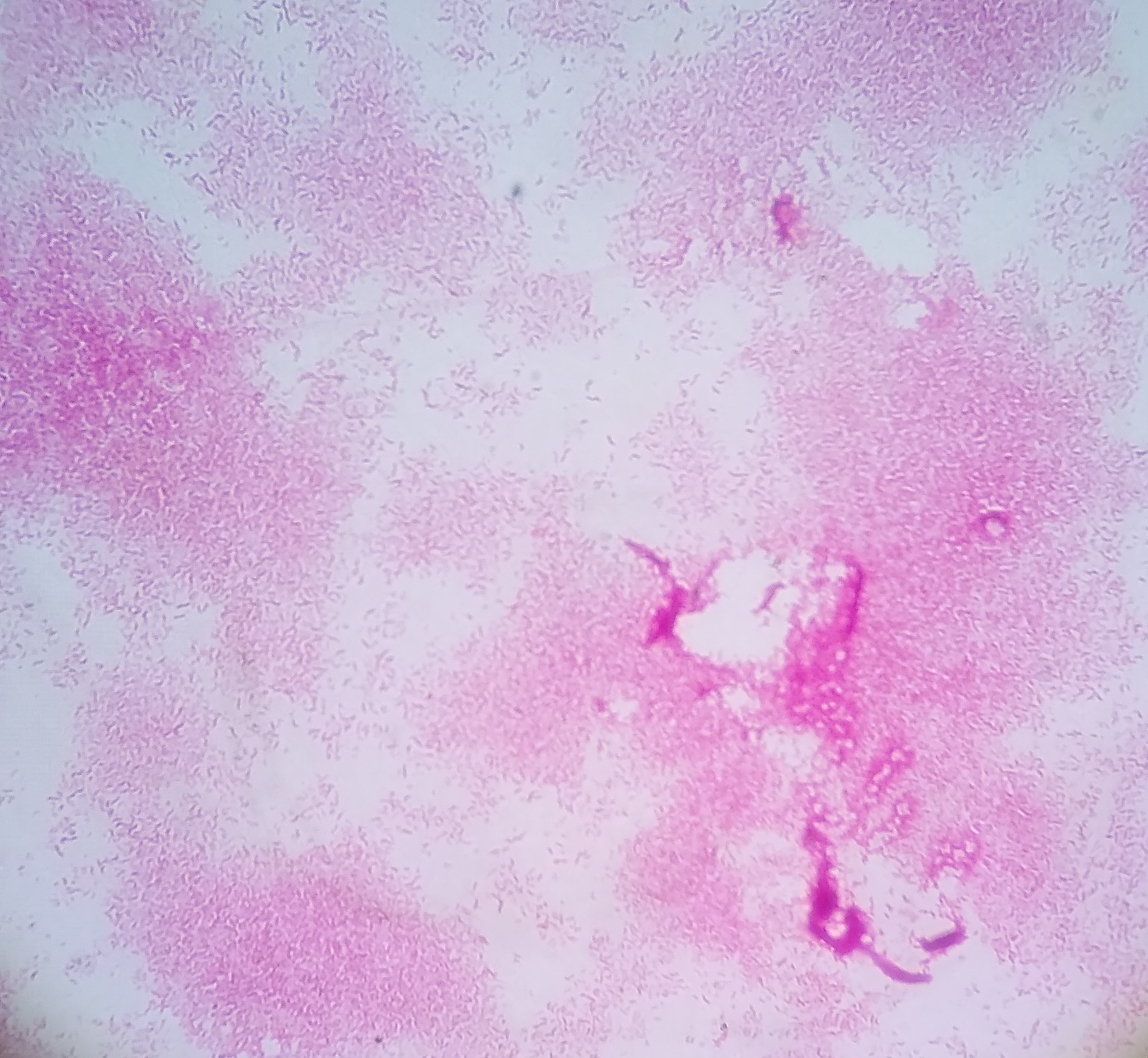

Supplement: Supplementary file 6 — Additional file 6. [file 12917_2022_3260_MOESM6_ESM.jpg]
